# Changes in Hepatitis C Awareness in Different Disciplines During COVID-19

**DOI:** 10.5152/tjg.2022.21726

**Published:** 2022-10-01

**Authors:** Sezgin Barutçu, Çiğdem Yıldırım, Abdullah Emre Yıldırım, Buğra Tolga Konduk, Zeynel Abidin Sayiner, Murat Taner Gülşen

**Affiliations:** 1 Department of Gastroenterology, Gaziantep University Faculty of Medicine, Gaziantep, Turkey; 2 Department of Internal Medicine, Gaziantep University Faculty of Medicine, Gaziantep, Turkey; 3 Department of Endocrinology and Metabolic Diseases, Gaziantep University Faculty of Medicine, Gaziantep, Turkey

**Keywords:** Awareness, COVID-19, hepatitis C

## Abstract

**Background:**

We aimed to determine the awareness of referring hepatitis C virus patients to the relevant departments and the effect of the pandemic period on this subject.

**Methods:**

A total of 65 743 patients with anti-hepatitis C virus requests before and during the COVID-19 pandemic were retrospectively screened. Anti-hepatitis C virus-positive patients were divided into 5 groups according to age distribution. The distribution of patients with anti-hepatitis C virus positivity was compared according to age groups, before and during COVID-19. Anti-hepatitis C virus-positive patients who were not requested hepatitis C virus RNA were evaluated individually according to the departments, and hepatitis C virus awareness was compared before and during COVID-19.

**Results:**

Anti-hepatitis C virus positivity rate was 1.54% before COVID-19; this rate was 2.15% during COVID-19. When the anti-hepatitis C virus positivity rate was compared in terms of age distribution according to before and during COVID-19, it was observed that there was a statistically significant decrease in the >65 age group in the COVID-19 period (*P *= .004). It was found that 216 (32%) of the patients who had anti-hepatitis C virus (+) before COVID-19 and 231 (48.1%) of the patients during COVID-19 were not requested hepatitis C virus RNA test (*P *< .0001). The departments with the highest awareness of hepatitis C virus were gastroenterology, infectious diseases, hematology, gynecology and obstetrics, and oncology, while the departments with the lowest hepatitis C virus awareness were ophthalmology, psychiatry, and general surgery. It was found that chronic hepatitis C virus awareness decreased in all departments during COVID-19.

**Conclusion:**

Hepatitis C virus awareness has decreased in all medical departments despite the physician alert system during COVID-19 and also the rate of anti-hepatitis C virus (+) patients decreased in the group aged >65 years during the pandemic.

## Introduction

Hepatitis C virus (HCV) infection is an important public health problem due to its high risk of chronicity and advanced complications such as cirrhosis and hepatocellular carcinoma. It is estimated that around 170-200 million people worldwide are infected with HCV.^1^ Acute HCV infection becomes chronic around 80%-85%, and 15%-20% of chronic HCV patients can progress to liver cirrhosis within 20 years.^2,3^

The worldwide prevalence of anti-HCV is estimated to be around 1.3%-2.1%. However, approximately 115 million people are thought to be infected with HCV. While most of these infections are seen in adults (104 million), the estimated prevalence of anti-HCV is around 2.0% (1.7-2.3%). The prevalence of viremic (HCV RNA positive) patients in adults is around 1.4% (1.2-1.7%) and it is estimated that there are approximately 75 million viremic patients.^4,5^ According to the first Global Hepatitis Report which was published by the World Health Organization (WHO) and 2015 global data, only 20% (5.7-36.3%) of patients were diagnosed and 7.4% (2.2-12.1%) were treated.^6^ In addition, according to the WHO data, less than 5% of people with chronic HCV infection are aware of their condition.

Although there are effective treatments for HCV, patients cannot access available therapies due to the lack of awareness about the diagnosis.^7^ According to Centers for Disease Control and Prevention data, the number of deaths due to HCV infection in 2015 (5 deaths/100 000) increased according to 2012 data (4.9 deaths/100 000) but decreased again in 2016 to 4.5 deaths/100 000.^8^ The aim of the WHO is to eradicate HCV completely with effective screening and treatment programs by 2030.^9^ However, current awareness must be increased for HCV eradication. In addition to raising the awareness of the patients, raising the awareness of their doctors plays a key role here.

The coronavirus disease (COVID-19), declared as a pandemic by the WHO, seriously strains health systems in all countries.^10^ Centers for Disease Control and Prevention has published guidance on postponing non-emergency procedures and routine outpatient checkups to reduce the healthcare burden throughout the COVID-19 pandemic.^11^ This has resulted in COVID-19 disrupting many cancer screening programs and slowing the hepatitis elimination program. The delay in the diagnosis and treatment of hepatitis is thought to result in approximately 44 800 liver cancer and 72 300 HCV-related deaths by 2030.^12^

For this purpose, we aimed to determine the awareness of referring HCV patients to the relevant departments and the effect of the pandemic period on this subject.

## Materials and Methods

Patients over the age of 18 who were admitted to Gaziantep Şahinbey Research and Application Hospital between March 2019 and March 2021, the period covering 1 year before and after the official acceptance of the COVID-19 pandemic in our country, were included in this study. Between these dates, patients who were requested an anti-HCV test were retrospectively screened. The demographic characteristics such as age and gender of the patients with positive anti-HCV test and the name of the department for which an anti-HCV test was requested were recorded from the hospital data processing center. For the same patient with more than 1 anti-HCV result, only the first positive test was included in the analysis. Anti-HCV-positive patients were divided into 5 groups as 18-35 years old, 35-45 years old, 45-55 years old, 55-65 years old, and >65 years old. The distribution of patients with anti-HCV positivity was compared according to age groups, before and during COVID-19. Among the patients with anti-HCV positive, those receiving chronic HCV treatment were excluded from the study. After these patients were excluded, the patients who were anti-HCV positive but did not have the HCV RNA test were evaluated according to the departments. These patients, who were anti-HCV positive but did not request HCV RNA, were compared according to the departments as before and during COVID-19. The study was performed in accordance with good clinical practice, and the Declaration of Helsinki and was approved by the local ethical committee (Ethical Committee of Gaziantep University Clinical Research, 2021/193).

### Anti-HCV and HCV RNA test methodology

Anti-HCV antibodies were measured with the ARCHITECT (Abbott Diagnostics Germany/Wiesbaden) device, which qualitatively detects immunoglobulin G and immunoglobulin M antibodies against HCV in human serum and plasma. It is a test based on the principle that the color change that occurs after combining recombinant HCV antigens with anti-HCV antibodies from the patient’s sample is detected by the optical system of the device. HCV RNA tests were performed on the real-time polymerase chain reaction (PCR) device (Roche Cobas Amliprep/Cobas Taqman 96 Germany) using the Abbott Real-Time HCV kit and reverse transcription PCR. After detecting the presence of HCV RNA in the sample, the HCV RNA level was quantitatively determined by real-time PCR.

### Statistical Analysis

The chi-square test was applied to compare independent proportions. Frequencies and percentages were given as descriptive statistics. Statistical analysis was performed with Statistical Package for the Social Sciences Statistics for Windows version 22.0 (IBM Corp.; Armonk, NY, USA) and a *P* <.05 was accepted as statistically significant.

## Results

Anti-HCV positivity was detected in 1154 (1.75%) of the patients who underwent 65 743 anti-HCV tests in total. While 43 520 anti-HCV test requests were made between March 2019 and March 2020 before the COVID-19, anti-HCV positivity was detected in 674 (1.54%) of the patients. Between March 2020 and March 2021, during the COVID-19 period, 22 223 anti-HCV test requests were made and anti-HCV positivity was detected in 480 (2.15%) of these patients. Regarding the gender distribution, 669 (58%) of the 1154 patients with anti-HCV positivity were women.

**Figure 1.**
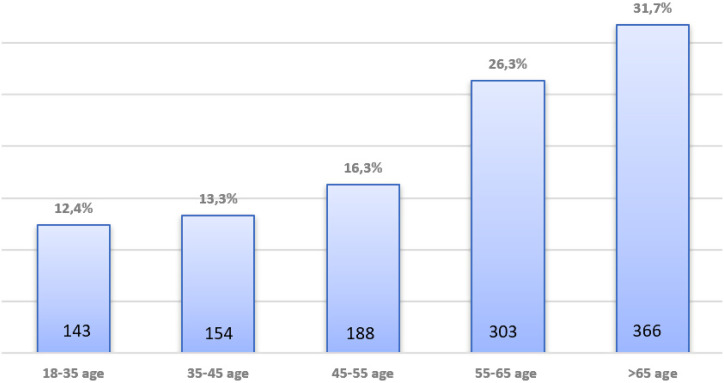
Age distribution of anti-HCV (+) patients. HCV, hepatitis C virus.

When the patients were classified according to age distribution, 143 (12,4%) were 18-35 years old, 154 (13,3%) were 35-45 years old, 188 (16,3%) were 45-55 years old, 303 (26,3%) were in the 55-65 age range, and 366 (31.7%) were over 65 years old (Figure 1). When the age distribution of the patients with anti-HCV positivity was compared in terms of the before COVID-19 and during COVID-19 periods, it was observed that there was a statistically significant decrease in the >65 age group during the COVID-19 period (*P *= .004). However, no statistically significant difference was observed in other age groups (Table 1).

It was found that 216 (32%) of the patients who had anti-HCV (+) before COVID-19 and 231 (48.1%) of the patients during COVID-19 were not requested for an HCV RNA test. There was a statistically significant difference between the 2 groups (*P *< .0001). Although there are warning systems that automatically alert if anti-HCV (+) is detected in our hospital, it was found that the rate of HCV RNA requests decreased during the COVID-19 period.

**Figure 2.**
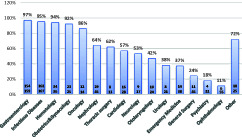
HCV RNA request rates in patients with anti-HCV (+) according to departments before COVID-19. HCV, hepatitis C virus.

**Figure 3.**
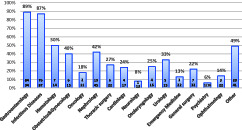
HCV RNA request rates in patients with anti-HCV (+) according to departments during COVID-19. HCV, hepatitis C virus.

Hepatitis C virus RNA request rates in patients with anti-HCV (+) according to departments before COVID-19 were 97% in gastroenterology, 95% in infectious diseases, 94% in hematology, 92% in obstetrics and gynecology, 86% in oncology, 64% in nephrology, 62% in thoracic surgery, 57% in cardiology, 53% in neurology 42% in otolaryngology, 38% in urology, 37% in emergency medicine, 24% in general surgery, 18% in psychiatry, and 11% in ophthalmology. When chronic HCV awareness was evaluated according to departments, this rate was found to be highest in gastroenterology, infectious diseases, hematology, obstetrics and gynecology, and oncology before COVID-19. The departments with the lowest awareness of chronic HCV were determined as ophthalmology, psychiatry, and general surgery, respectively (Figure 2). Although chronic HCV awareness decreased in almost all departments during the COVID-19 period, the highest drop was found in hematology, oncology, and obstetrics and gynecology (Figure 3). It was determined that the awareness of chronic HCV decreased statistically significantly in the departments of gastroenterology, infectious diseases, hematology, obstetrics and gynecology, oncology, nephrology, thoracic surgery, cardiology, neurology, and emergency medicine after the COVID-19 pandemic (Table 2).

## Discussion

The new coronavirus severe acute respiratory syndrome coronavirus 2, which causes COVID-19 in late 2019, was declared as a global emergency by the WHO. This issue has placed a significant burden on national health systems.^13^ The Global Health Sector Strategy is to eradicate HCV by 2030. However, with the COVID-19 pandemic, this period may be extended. During the early stages of the COVID-19 pandemic, most non-emergency healthcare services had to be postponed in many countries due to the increased burden on the healthcare system.^14^ One of the important factors in predicting whether this process will be prolonged may be attributed to the alteration in the focus of clinicians dealing with hepatitis C in the diagnosis and treatment process of HCV.^15^

In our study, we examined whether there was a change in the implementation of the hepatitis C diagnosis and treatment program in one of the most important treatment centers in the region. It was also found that the rate of HCV RNA requests decreased during the COVID-19 period despite the fact that there are warning systems that automatically alert if anti-HCV positivity is detected in the hospital. Disruptions during HCV treatment have been reported in many countries during the pandemic. Disruption in the diagnosis and treatment of HCV continue to be reported in various countries with very different pandemic conditions.^16^ In a study, it was reported that a delay of at least 1 year in the HCV eradication program at best is predicted.^12^ The National Health and Nutrition Examination Survey (NHANES) study of 30 140 participants showed insufficient awareness of HCV infection among physicians.^17^ In a retrospective study based on data from a single-center, tertiary hospital between 2000 and 2017, physicians’ awareness of HCV testing in Turkey was found to be approximately 50% in the last decade.^18^ In our study, while the above-mentioned rate was inconsistent with this study before the pandemic (48.1%), this rate decreased significantly during the pandemic (32%).

The distribution of patients with anti-HCV (+) detected within themselves was analyzed before and during the pandemic. It was noteworthy that the rate of anti-HCV (+) patients decreased significantly in the group aged >65 years during the pandemic compared to the pre-pandemic period. During the pandemic process, states follow different restriction policies. This outcome may be due to the long-term curfew of people over the age of 65 in Turkey. We think that curfews reduce hospital admissions. In addition, continued caution is required in testing individuals born between 1945 and 1965 (Baby boomers) who are at the highest risk of dying from hepatitis C-related causes.^19^ We think that this decrease in the test rates of people over 65 years old poses a serious risk for the development of HCV complications. There are studies showing that delayed diagnosis of hepatitis C also causes a delay in HCV antiviral treatment.^20^ Moreover, delays in identifying individuals infected with hepatitis C will increase the potential transmission of HCV infection to others and, in the long run, increase the incidence of complications of the disease in untreated individuals.^21^

Effective novel antivirals used in the treatment of HCV have significantly decreased HCV-related complications and deaths in recent years. Although the success rate in treatment has increased in recent years, the inability to find HCV patients also prevents them from reaching treatment.^15,22^ Increasing the awareness of physicians as well as patients is very important in increasing HCV awareness. In our study, HCV awareness was found to be high before COVID-19 in gastroenterology, infectious diseases, hematology, obstetrics and gynecology, and oncology departments, while HCV awareness was found quite low in ophthalmology, psychiatry, and general surgery departments. In the COVID-19 period, it was put forth that HCV awareness decreased in all departments. We think that the heavy workload of the departments and the additional health burden brought by the COVID-19 pandemic have led to this outcome. In addition, the decrease in the time allocated to the patient during the COVID-19 pandemic may also be a confounding factor. While there are publications with similar awareness distributions as in our study, there are publications with opposite results.^18,23^ The lack of physician awareness causes delays in directing patients with anti-HCV positivity to the relevant departments. To summarize, the patient’s access to HCV treatment is delayed and HCV eradication becomes difficult. Although there is a physician warning system in our center, the decrease in awareness suggests that additional methods should be taken to increase HCV awareness.

In conclusion, HCV RNA request rates in patients with anti-HCV positivity detected during the pandemic period decreased significantly in all medical departments despite the physician warning system. The increase in workload during the pandemic period and avoidance of contact with the patient can be shown as the reason for this. The lack of directing the patients to the relevant departments indicates that additional precautions should be taken. Further studies are needed in this regard.

## Figures and Tables

**Table 1.** Comparison of Anti-HCV (+) Patients According to Age Groups Before and During COVID-19

	**Total Anti-HCV(+) Patients**	**18-35**	**35-45**	**45-55**	**55-65**	>65
**Before COVID-19**	674	74 (11%)	86 (12.7%)	101 (15)	177 (26.3%)	236 (35%)
**During COVID-19**	480	69 (14.4%)	68 (14.3%)	87 (18.1%)	126 (26.2%)	130 (27%)
*P*		.084	.302	.154	.997	**.004**

HCV, hepatitis C virus.

*P* <.05 is the signifant cut off point.

**Table 2.** Comparison of HCV Awareness Between Departments Before and During COVID-19

	**2019** **Anti-HCV (+) Patients Number (n)**	**2019** **Percentage of HCV RNA Testing (%)**	**2020** **Anti-HCV (+) Patients Number (n)**	**2020** **Percentage of HCV RNA Testing (%)**	*P*
**Gastroenterology**	159	97	94	89	**.009**
**InfectiousDiseases**	107	95	87	87	**.048**
**Hematology**	36	94	14	50	**.001**
**Obstetrics&Gynecology**	25	92	15	40	**.001**
**Oncology**	14	86	11	18	**.001**
**Nephrology**	45	64	45	42	**.037**
**Thoracic surgery**	13	62	22	27	**.044**
**Cardiology**	28	57	17	24	**.032**
**Neurology**	17	53	13	8	**.011**
**Otolaryngology**	24	42	16	25	.276
**Urology**	21	38	15	33	.761
**Emergency medicine**	38	37	22	13	**.048**
**General surgery**	45	24	32	22	.839
**Psychiatry**	22	18	16	6	.283
**Ophthalmology**	55	11	22	14	.715
**Other**	25	72	41	49	.068

HCV, hepatitis C virus.

*P* <.05 is the signifant cut off point.
